# Functional evaluation of a real-time EMG controlled prosthetic hand

**DOI:** 10.1017/wtc.2025.7

**Published:** 2025-04-07

**Authors:** Amlan Jyoti Kalita, Maibam Pooya Chanu, Nayan M. Kakoty, Ramana Kumar Vinjamuri, Satyajit Borah

**Affiliations:** 1 Embedded Systems and Robotics Laboratory, Tezpur University, Tezpur, India; 2 Vinjamuri Lab, University of Maryland Baltimore County, Baltimore, MD, USA; 3 TIMeS Hospital, Tezpur, India

**Keywords:** prosthetic hand, electromyogram, box and block test, pick and place test

## Abstract

Electromyogram (EMG)-controlled prosthetic hands have advanced significantly during the past two decades. However, most of the currently available prosthetic hands fail to replicate human hand functionality and controllability. To measure the emulation of the human hand by a prosthetic hand, it is important to evaluate the functional characteristics. Moreover, incorporating feedback from end users during clinical testing is crucial for the precise assessment of a prosthetic hand. The work reported in this manuscript unfolds the functional characteristics of an EMG-CoNtrolled PRosthetIC Hand called ENRICH. ENRICH is a real-time EMG controlled prosthetic hand that can grasp objects in 250.8 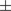 1.1 ms, fulfilling the neuromuscular constraint of a human hand. ENRICH is evaluated in comparison to 26 laboratory prototypes and 10 commercial variants of prosthetic hands. The hand was evaluated in terms of size, weight, operation time, weight lifting capacity, finger joint range of motion, control strategy, degrees of freedom, grasp force, and clinical testing. The box and block test and pick and place test showed ENRICH’s functionality and controllability. The functional evaluation reveals that ENRICH has the potential to restore functionality to hand amputees, improving their quality of life.

## 1. Introduction

Upper limb amputation is a life-changing event for an individual, with substantial functional and occupational implications. In the past two decades, various research studies have been conducted on enhancing the capabilities and realism of prosthetic hands (Azocar et al., 2020). While these advancements have benefited some amputees, the utilization of prostheses remains limited (Nagaraja et al., 2016; Jansen, 2020). Users’ perceptions towards limited use of prosthetic hands often cite reasons such as physical discomfort, low functionality, or high cost (Walker et al., 2020; Resnik et al., 2022; Gentile and Gruppioni, 2023). Given the user-specific nature of prosthetic hands, research on prostheses must incorporate users’ feedback during clinical testing (Jansen, 2020). Unfortunately, although many of the existing research prototypes of prosthetic hands may have the potential to be useable, many of these research prototypes have been tested without clinical evaluation. Thus, these research versions fall short of achieving user satisfaction in real-world scenarios (Atasoy et al., 2018; Abarca et al., 2019; Azocar et al., 2020). Additionally, commercially available advanced prosthetic hands, such as Taska hand (Prosthetics, 2022), bebionic (Ottobock, 2020) and i-limb (Össur, 2021), are comfortable and have high functionality but are prohibitively expensive. Consequently, there is a need to assess the functional characteristics of prosthetic hands to facilitate better adaptation and coexistence with users.

The anthropomorphism of prosthetic hands can be assessed through various human hand attributes (Belter and Dollar, 2011). Prosthetic hand users have a wide range of requirements, including functionality, comfort, ease of use, aesthetics, durability, and support (Cordella et al., 2016). Functionality includes the ability to carry out essential daily activities, supported by a design that incorporates sufficient degrees of freedom (DoF) and range of motion (RoM) to closely mimic the movements of human hands. Comfort and fit are critical, necessitating a personalized acceptance. Further, user acceptance is significantly influenced by the weight of a prosthetic hand, considering that the human hand typically accounts for 5.8% of total body weight (Krishnan et al., 2016). A lightweight design is needed to reduce fatigue during the use of a prosthetic hand (Ryu et al., 2020). Notably, many existing prosthetic hands tend to be excessively heavy for users (Belter and Dollar, 2011). For ease of use, a prosthetic hand should have intuitive control and minimal training (Mohammadi et al., 2020). Users’ feedback is critical for continual improvement and offering emotional and practical assistance to users (Sensinger and Dosen, 2020). A prosthetic hand addressing the above issues will result in a functional hand, satisfying the users requirement. Therefore, functional characteristics of prosthetic hands should consider DoF, RoM, dimension, and weight in comparison to the human counterpart. In addition, the size of prosthetic hands should be a mirror to the natural counterparts, as aesthetics should focus on a natural appearance (Panero et al., 1979). The interface between the prosthetic hand and user is crucial for users’ acceptability (Li and Felländer-Tsai, 2021). Prosthetic hands with a higher number of DoF facilitate daily activities, and the RoM in finger joints is essential to mimic the capabilities of the human hand (Barakat et al., 2013; Prudencio et al., 2014). A prosthetic hand with operation time that satisfies neuromuscular constraints is identified as another essential characteristic of upper limb prostheses. Existing prosthetic hand prototype often excel in one or two key attributes but fail to replicate natural functionality, limiting their effectiveness and user satisfaction in real-world settings. To address this, prosthetists should integrate user feedback into the design and development process to create more acceptable prosthetic solutions.

Belter and Dollar (2011) conducted a comprehensive review of the performance characteristics of both commercial prostheses and research prototypes. The assessment involved evaluating various hand attributes and reporting comparisons among prosthetic hands. However, their analysis did not incorporate a comparison with the human counterpart. In a different approach (Kakoty and Hazarika, 2013), a biomimetic similarity index was introduced to express the likeness between prosthetic and human hands. The developed framework can quantify the anthropomorphism of upper limb prostheses using formal concept analysis. Despite the thoroughness of this work, it did not consider the speed of operation concerning neuromuscular constraints. Neuromuscular constraint refers to the duration required to perform a grasping operation from the initiation of the thought process at the neuronal level (Kakoty et al., 2022; Jerde et al., 2003). Notably, none of the mentioned studies incorporated clinical testing. However, understanding end-user responses is critical for establishing performance requirements for prosthetic hands intended for daily living activities (Azocar et al., 2020). It is worth noting that limited reports on clinical testing of developed prototypes have been documented till date (Pons et al., 2004; Zhang et al., 2011; Hargrove et al., 2017; Azocar et al., 2020).

Clinical testing of prosthetic hands is essential to evaluate their functionality and effectiveness in restoring lost limb capabilities (Pons et al., 2004; Zhang et al., 2011; Calli et al., 2015; Hargrove et al., 2017; O’Brien et al., 2022). It characterizes the usability of the prosthetic device, focusing on how well users can integrate the prosthetic hand with daily activities. To assess a prosthetic hand, different standard methods like the Box and Block Test (BBT) (Hashim et al., 2021), Nine-Hole Peg Test (NHPT) (Haverkate et al., 2016), Assessment of Capacity for Myoelectric Control (ACMC) (Lindner et al., 2009), Southampton Hand Assessment Procedure (SHAP) (Resnik et al., 2023), Anthropomorphic Hand Assessment Protocol (AHAP) (Llop-Harillo et al., 2019), and so forth were used. BBT assesses dexterity by measuring the number of blocks that an individual can transport from one compartment to another within a minute using the prosthetic hand. The test evaluates the user’s ability to manipulate small objects, which is critical for daily tasks. BBT directly addresses users’ insights into prosthetic hand use by picking up, moving, and placing blocks, which requires precise control and coordination. SHAP and AHAP are comprehensive but involve more complex tasks that require multiple grasp patterns (e.g., pinch, tripod, lateral, spherical, and so forth). Therefore, the BBT provides valuable, efficient, and standardized insights into the usability of prosthetic hands, justifying its sufficiency in the evaluation of the reported prosthetic hand. The NHPT is another process for measuring fine motor skills as individuals place and remove pegs from holes, providing information on hand coordination and speed (Haverkate et al., 2016). A similar method is followed to conduct the pick and place test, which is detailed in Section 3.2. It is significant to consider the hand assessment procedure to evaluate the functional characteristics of a prosthetic hand. Various studies were published to evaluate the efficacy of these tests. However, a few researchers have included hand assessment tests as a part of the development process of their prosthetic hand prototypes.

The work reported in this manuscript focused on assessing the functional evaluation of a prosthetic hand known as the EMG-coNtrolled pRosthetIC Hand (ENRICH), comparing it with existing commercial variants and laboratory prototypes vis-à-vis the human hand. The work reported in this manuscript is an extension of the work presented at the International Conference on Advances in Robotics 2023 and is available at https://dl.acm.org/doi/abs/10.1145/3610419.3610444. The previous work compared ENRICH with three commercial variants and 13 laboratory prototypes of prosthetic hands. Various hand attributes were considered for the evaluation, including weight, size, integration socket, DoF, finger joint RoM, control strategy, operation time, and clinical testing. While earlier research evaluated ENRICH as one of the promising hands, details of critical evaluation on design and clinical testing were not reported. Further, the discussions on various attributes (e.g., socket, grasp force, and so forth) were limited. These discussions, along with a critical evaluation of design and clinical testing, are crucial for the completeness of the functional evaluation leading to the users’ acceptability of ENRICH. These insufficiencies in the previous work motivated the extension and report a comprehensive approach to the design, development, and functional evaluation of ENRICH. The current state-of-the-art lacks a complete evaluation of design attributes with clinical testing for prosthetic hand evaluation. This manuscript addresses this gap, as it is essential for the users’ acceptability. This manuscript also comprises information on the design and development process of ENRICH, control strategy, laboratory testing, standard prosthetic hand evaluation testing (BBT and pick and place tests), and comparison with 10 commercial variants and 26 laboratory prototypes. The extended works comprise details of design (in Section 2), initial laboratory testing (in Section 2.1), BBT (in Section 3.1), pick and place test (in Section 3.2), interfacing socket (in Section 4.1.3), control strategy (in Section 4.2.1), and grasp forces (in Section 4.2.3). In succinct, this manuscript presents the process of prosthetic hand design, development, and functional evaluation, incorporating feedback from end-users. Thus, ENRICH takes a significant step in replicating human hands with tangible benefits for upper limb amputees. The manuscript is organized as follows: in Section 2, the design of ENRICH is reported. In Section 3, the clinical testing, including BBT and pick and place tests is reported. In Section 4, the functional characteristics of ENRICH in terms of physical properties, grasping properties, and kinematic properties are described. A comparison of ENRICH with other prosthetic hands was presented in Section 5. The concluding remarks are presented in Section 6.

## 2. Design of ENRICH

Improving the acceptability of prosthetic hands focuses on creating devices that users are more willing to embrace, which can lead to improved quality of life. This can be achieved by keeping the users’ input into the design and development of a prosthetic hand. This motivation was followed while designing ENRICH, an upper limb prosthesis device controlled in real-time through EMG. The target performance for ENRICH was to emulate the speed, dexterity (RoM and DoF), strength, weight, and size of a human hand. Achieving human-like performance enhances the prosthetic hand’s natural feel and usability.

**Figure 1.**
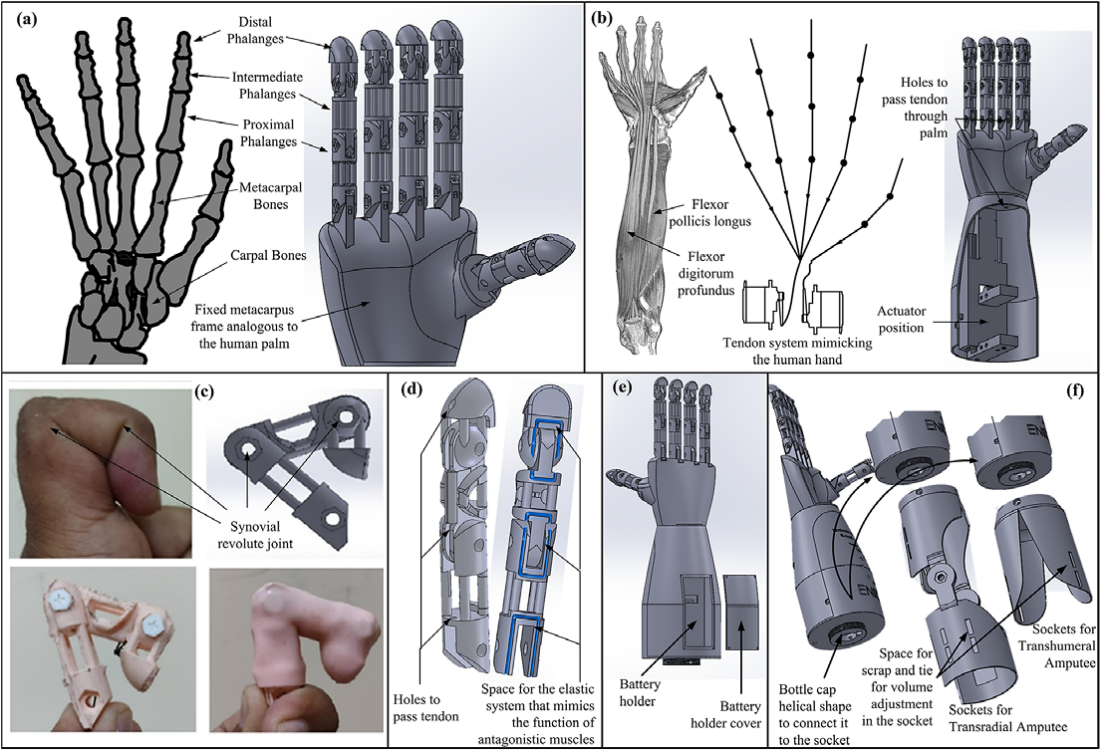
(a) CAD of the ENRICH, showcasing its biomimetic adaptation of the human hand. (b) Tendon routing inspired by human anatomy, highlighting flexion extension mechanisms and actuator position. (c) Synovial revolute joint design for a finger’s naturalistic motion replication. (d) Finger structure with spaces for tendon and elastic systems mimicking agonist and antagonistic muscle function. (e) Dorsal view of ENRICH with the battery compartment. (f) Modular sockets for transhumeral and transradial amputees, showcasing adaptability and volume adjustment features.

The computer-aided design (CAD) of ENRICH, a five-fingered prosthetic hand, was developed to mimic the natural counterpart as shown in Figure 1(a). Terminologies from human anatomy had been employed to characterize the CAD of ENRICH. The distal, middle, and proximal phalanges of a human (Jones and Lederman, 2006) finger were replicated by three links on each finger as presented in Figure 1(a). The thumb was made up of two links. Maintaining a lightweight design ensures that the prosthetic hand does not place unnecessary strain on the user’s residual arm (Milfont and Gómez-Malagón, 2021). This contributes to long-term wearability and ease of use. To optimize the weight, minimum solid parts were kept in the CAD of fingers, maintaining the overall shape as shown in Figure 1(c). Revolute joints that correspond to the distal interphalangeal (DIP), proximal interphalangeal (PIP), and metacarpophalangeal (MCP) joints (Jones and Lederman, 2006) were used to connect the links. These joints were developed to mimic finger anatomy’s synovial revolute joints (Marquez-Florez et al., 2022) by customizing the anterior end of finger links. The ends of the cylindrical finger links were cut in a quarter-circular form on the anterior side of the finger, with a radius of curvature equal to half the finger thickness. The quarter circle carve in the posterior section was not constructed to limit finger mobility beyond hyperextension (Prudencio et al., 2014). These enabled ENRICH to mimic human finger flexion and extension. Human palms typically include five bones known as metacarpal bones (Jones and Lederman, 2006). To reduce mechanical complexity, the palm was designed with a fixed metacarpus frame analogous to the human palm, as presented in Figure 1(a).

The forearm includes all the actuators (TowerPro MG958 Servo Motor) and control and power regulation circuits. Flexor digitorum profundus (FDP), flexor pollicis longus (FPL), and extensor digitorum communis (EDC) muscles are part of the forearm flexor-extensor muscles, which are primarily responsible for the grasping by the human fingers (Sabapathy et al., 2005). To replicate human anatomy, actuators were deliberately placed in the forearm section of ENRICH. Servo motors were chosen for their ability to provide precise control over position and speed. Additionally, they can replicate the back-and-forth motion, similar to the agonist and antagonist muscle pairs in humans, enabling flexion and extension movements (Belanger et al., 1983). ENRICH employs two actuators: one dedicated to the thumb and the other to the remaining four fingers. This design mirrors the natural arrangement of human flexion-extension (as presented in Figure 1(b)), where the FDP muscle controls the four fingers, and the FPL muscle controls the thumb (Drake et al., 2009). It is an efficient and effective approach to create a functional yet simplified model. The battery (7.4 V, 1,000 mAh, Li-Polymer) was placed in a rectangular cuboid cavity located on the dorsal side of the forearm, as shown in Figure 1(e). This placement ensures easy access for the user, allowing for convenient charging or replacement. A natural size is critical for aesthetic reasons and for interacting effectively with objects. The details of size considered are further discussed in Section 4.1.1.

To transmit force from actuators to fingers, transmission systems such as bar mechanisms and pneumatic systems are used to provide strong and precise control (Taniguchi et al., 2020; Wahit et al., 2020). However, these systems are often bulky and heavy. An alternative approach involves using smart materials like shape-memory alloys, which eliminate the need for traditional mechanical transmission systems (Hamid et al., 2023). While promising, this method typically suffers from slower response time and lower force output. In comparison to these systems, tendon-driven systems resemble how muscles control bones in the human hand. These systems can be highly effective and lightweight. Following this, an active human biomechanics-inspired tendon mechanism (Ozawa et al., 2013) was implemented for the flexion of fingers. The tendon system of ENRICH was based on the agonist-antagonistic tendon mechanism found in the human hand. In this mechanism, one muscle contracts while the opposing muscle lengthens. In ENRICH, tendons were routed through holes that were made at the ventral side of fingers and tied to the tips of the fingers, as illustrated in Figures 1(b) and (d), to generate torques at the joints. The tendon passed through the fingers and palm. The other end of the tendon was tied to the actuator. An elastic system, incorporated into the MCP, PIP, and DIP joints (as shown in Figure 1(d)), mimics the function of antagonistic muscles. When actuated, the tendons on the ventral side shorten across the actuator, producing finger flexion. During this process, the antagonistic system on the dorsal of the finger lengthens. Finger extension occurs when the actuator releases the tendon, and the elastic system pulls the finger back to its original position.

ENRICH is adaptable for both below-elbow amputees (transradial amputee) and above-elbow amputees (transhumeral amputees), with corresponding sockets as shown in Figure 1(f). The inner wall of the forearm was designed like a bottle cap helical shape to connect it to the socket. Volume adjustment belts allow users to tie the socket in their remnant muscle according to their convenience.

Using additive manufacturing, the 3D parts of the prosthetic hand were developed. Various materials can be used for 3D printing such as polylactic acid (PLA), acrylonitrile-butadiene-styrene (ABS), nylon, glycol-modified PET (PETG), thermoplastic polyurethane (TPU), etc. (Gregor-Svetec, 2022). PLA is made from renewable resources such as corn starch (Fatchurrohman et al., 2023), making it an eco-friendly option. It can also biodegrade in industrial composting facilities (Fogašová et al., 2022). It also has a low melting point (Avinc and Khoddami, 2009), making it easier to print than other materials. Further, PLA is more affordable, making it a good choice for 3D-printed prosthetic hands. Considering these benefits, PLA was selected for the additive manufacturing of ENRICH.

The design of ENRICH stands out for its biomimetic approach, emphasizing not only functional performance but also user-centric considerations. Unlike conventional prosthetic hand designs that focus on a single target performance, ENRICH integrates critical attributes such as speed, dexterity (RoM and DoF), strength, weight, and size to closely replicate the human hand. Crucial aspects, such as the dimensions of the phalanges, the placement of actuators, the selection of actuators, and the choice of transmission mechanisms, were carefully chosen to emulate the anatomy of the human hand. Most notably, ENRICH incorporates iterative feedback from clinical testing, ensuring its design aligns with the practical needs and preferences of end users, setting a new standard for acceptability and usability in prosthetic hands.

### 2.1. Initial testing

**Figure 2.**
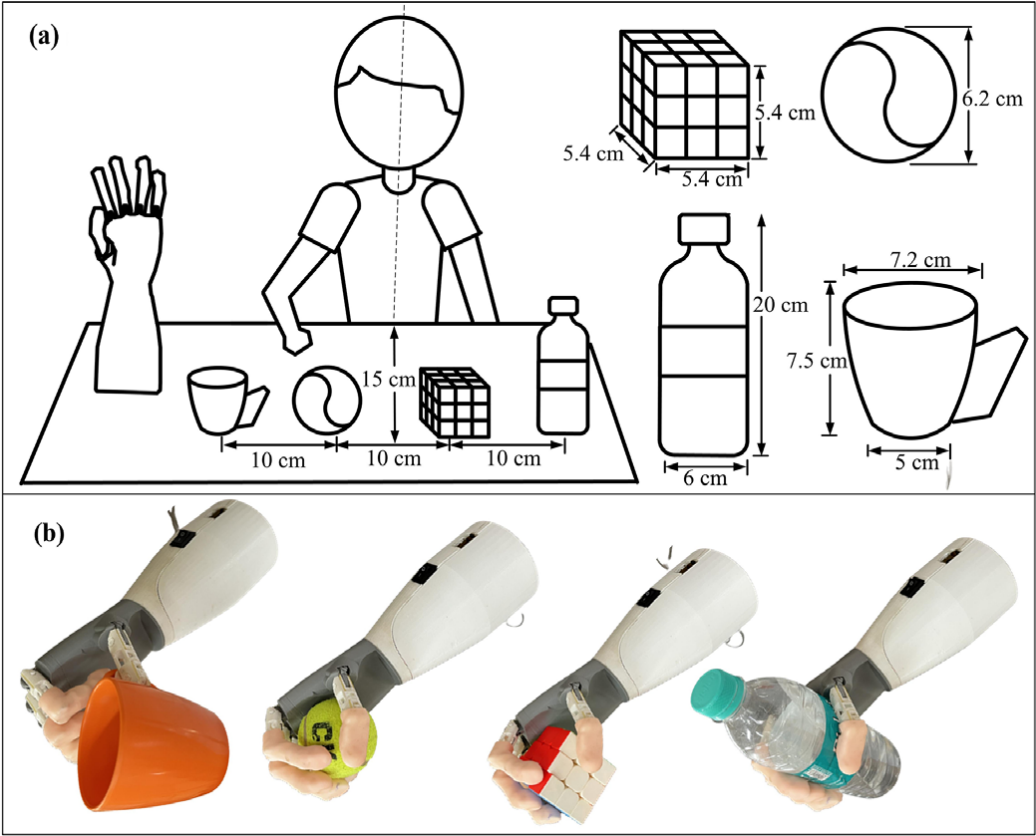
(a) Experimental setup and dimension of the grasping objects. (b) Grasping of four objects by ENRICH.

Prior to the clinical testing, ENRICH was tested in a laboratory environment with EMG collected from healthy subjects. Four objects, a coffee mug (70 g), a tennis ball (70 g), a Rubik’s cube (60 g) and a water bottle (450 g), were considered for grasping experiments. The dimensions of the objects are presented in Figure 2(a). The four objects were placed in a table, 10 cm apart and 25 cm away from the midline of the subjects as shown in the experimental setup in Figure 2(a). EMG electrodes were placed on the trapezius muscle of the subject to control the prosthetic hand ENRICH. At the start of the experiment, the subjects were asked to express the grasping intention by generating EMG from the targeted muscle. On detection of grasping intention by the controller embedded into the hand, ENRICH lifts the grasping objects and places them back in the same position. The experiments were repeated for 10 trials for each object. It was observed that the subject could grasp and place the object successfully during each trial. No objects fell or slipped during the experiment. Figure 2(b) shows grasping the four objects by ENRICH.

## 3. Clinical testing

Clinical testing of ENRICH was carried out with eight amputees. It was conducted with the ethical consent of Tezpur Institute of Medical Sciences Hospital, Tezpur, with approval number THEC/2022/B-03. The participants (five males and three females) aged 26.67 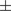 7.04 years took part in the experiment. The clinical testing of ENRICH yielded an average grasping accuracy of 98.25%. Users’ subjective inputs were received on the weight, ease of use, grasping power, and effort to manage grasping. The visual appeal of the ENRICH was taken into account while reviewing and redesigning it. The redesigned hand was then provided to three amputees for pilot testing to ensure its viability and resilience in a real-world context. They were using ENRICH daily to perform their day-to-day activities. To assess ENRICH, BBT and pick and place tests were performed with a known user. Tasks under these tests are considered representative of daily living activities (Haverkate et al., 2016; Hagovska and Nagyova, 2017).

### 3.1. Box and block test

BBT is a quantifiable test that provides a measure of an individual’s upper extremity functionality (Salminger et al., 2019). This is crucial for assessing motor skills required for daily activities. By tracking the number of blocks transferred within 60 s from one place to another, the BBT score offers a clear benchmark for evaluating and hand functionality monitoring progress over time (Hashim et al., 2021; Siegel et al., 2023). Additionally, the standardized nature of the BBT score facilitates consistent comparisons across different individuals, making it a valuable tool for clinical testing (Salminger et al., 2022).

#### 3.1.1. Test setup

**Figure 3.**
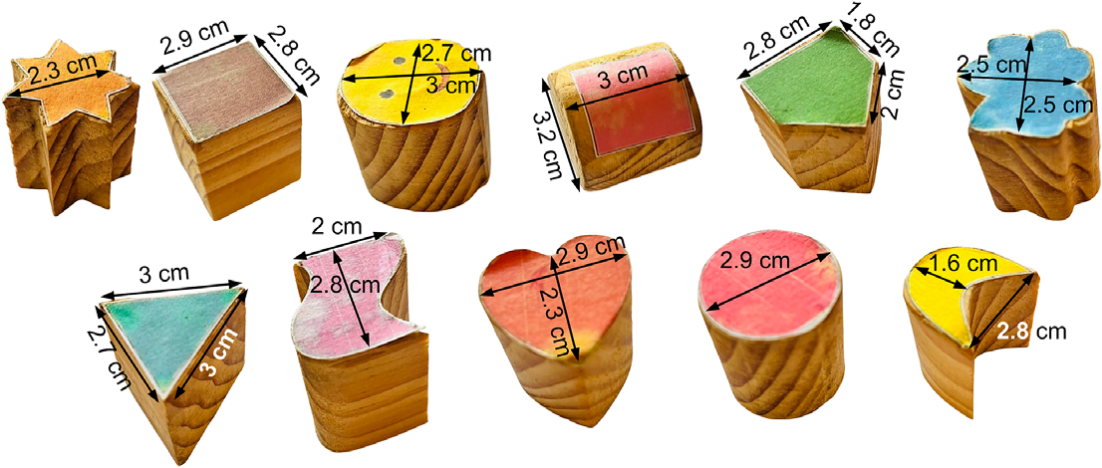
Blocks used in the BBT and pick and place test.

The BBT test setup comprises a box with two compartments (Compartment I and Compartment II), with a partition and 11 blocks. Table 1 presents the dimensions of the BBT setup. The blocks to be transferred from Compartment I to Compartment II during the test were differently shaped, like cylindrical, cubic, stared, triangular prism, heart-shaped, crescent, and pentagonal as presented in Figure 3. This is to ensure that the prosthetic hand, that is ENRICH under testing, can grasp differently shaped objects.

**Table 1.** Box and block test setup dimensions (in cm)

	Length	Width	Height
Compartment I	21	21.5	7
Compartment II	21	21.5	7
Partition	0.2^a^	21.5	7
Blocks	^b^	^b^	3.4

a Partition is made with a single cardboard.

b Blocks lengths and widths are represented in Figure 3.

#### 3.1.2. Subject

A female subject, aged 27, with transhumeral amputation, volunteered for the experiment. Her height measured 135 cm, and her weight was 40 kg. The amputation was a result of an accident that occurred one year ago from the date of day 1 of BBT. ENRICH was fitted to the subject’s residual limb through a socket. A single-channel Ag/AgCl surface electrode was positioned on the trapezius muscle, and the reference electrode was placed on the collarbone. On expression of grasping intention by the subject via voluntary contraction of the trapezius muscle, ENRICH performs grasp open and close following the control strategy explained in Section 4.2.1.

The study’s inclusion criteria required amputees who regularly used ENRICH and had no visual and hearing impairments. This criterion was crucial as the subject’s visual feedback was necessary for the successful completion of the experiment (Hashim et al., 2021; Kakoty et al., 2022).

#### 3.1.3. Experiment

**Figure 4.**
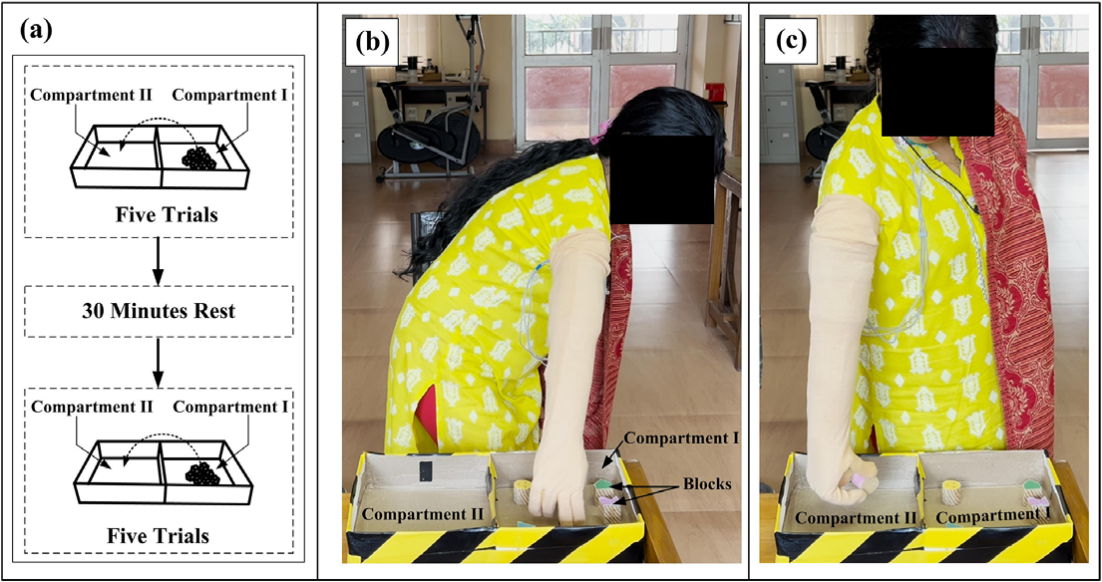
(a) The experimental protocol for the BBT test. (b) Blocks picked up from Compartment I. (c) Block successfully transferred to Compartment II.

The experiment was conducted with the subject in a standing position. The experimental protocol for the BBT is presented in Figure 4(a). All blocks were initially placed in Compartment I. The subject was instructed to move as many blocks as possible from Compartment I to Compartment II using ENRICH. Only one block can be moved at a time, and the hand must cross the partition to count as a successful transfer. The number of blocks successfully transferred to Compartment II in 60 s was recorded as the BBT Score. The test was conducted for 10 trials in two phases: five trials in Phase I and five trials in Phase II. A rest period of 30 min was added between the two phases. The inclusion of a rest period aims to prevent muscle fatigue resulting from continuous testing (Fang et al., 2022). BBT was completed in two experimental days, with day 2 occurring 10 days after day 1. Figures 4(b) and (c) show the subject involved in the BBT transferring objects from Compartment I to Compartment II.

#### 3.1.4. Experimental results

**Figure 5.**
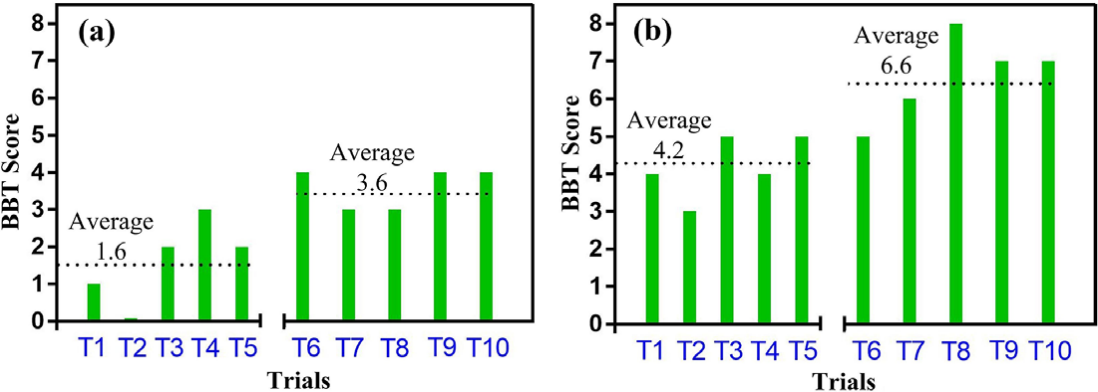
BBT score with respect to each trial on (a) day 1 and (b) day 2.

Table 2 presents the BBT score obtained from the BBT on day 1 and day 2. This score is the number of blocks transferred in 60 s from Compartment I to Compartment II. The average score of BBT was calculated to be 1.6 and 3.6 in Phase I and Phase II, respectively, during day 1 and 4.2 and 6.6 in Phase I and Phase II, respectively during day 2. The BBT scores of ENRICH with the average of each phase on day 1 and day 2 are presented in Figures 5(a) and (b), respectively.

**Table 2.** Box and block test score on day 1 and day 2

Phase	Phase I						Phase II				
Trials	T1	T2	T3	T4	T5	30 min rest	T6	T7	T8	T9	T10
BBT score on day 1	1	0	2	3	2		4	3	3	4	4
BBT score on day 2	4	3	5	4	5		5	6	8	7	7

From Figure 5, it was observed that the BBT score improved in Phase II compared to that of Phase I on both experimental days. Further, it was observed that the BBT score on day 2 as well also improved compared to that of day 1. The percentage of improvement in the average BBT score between Phase I and II and between day 1 and 2 was estimated using Equation (3.1).



(3.1)

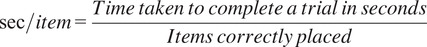




where, 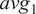 = average BBT score in Phase I

and 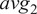 = average BBT score in Phase II.

The result shows a 55% improvement in the average BBT score in Phase II compared to that of Phase I on day 1. On day 2, a 36.37% improvement was observed in the average BBT score in Phase II compared to that of Phase I. These results indicate that the performance of ENRICH improves with practice. This is further confirmed by the improvement of 51.85% in the average BBT score on day 2 compared to that of day 1. The improvement in the BBT score indicates ENRICH’s functional capability increases over time with more practice. Subject’s visual feedback plays a crucial role in increasing hand-eye coordination during the BBT (Hill and Lindner, 2024). Initially, the subject identifies the object to be grasped and analyzes its shape, size, and orientation. The brain processes this information, determining the object’s position relative to the prosthetic hand. As the prosthetic hand reaches toward the object, the eyes provide continuous feedback, ensuring the trajectory is accurate. Fine adjustments are made based on visual cues to align the object correctly with the fingers of the prosthetic hand. Visual feedback also assists in confirming that an object is firmly grasped by the prosthetic hand. During transit, visual monitoring helps navigate around obstacles and maintain a stable path. Upon approaching the target location, the eyes assess the placement location, ensuring the object is positioned securely. This process is enabled by the effective controller of ENRICH that prevents slip through visual biofeedback (Kakoty et al., 2022; Choi et al., 2023). As a result, users can maintain a secure grasp on objects while moving the blocks from Compartment I to Compartment II. The result of BBT reflects ENRICH’s performance increases over time because of visual feedback, and therefore it can be beneficial for amputees to perform daily living activities effectively with practice over time.

### 3.2. Pick and place test

The pick and place test is significant in evaluating the object handling characteristics of a prosthetic hand (Khan et al., 2021). It assesses an individual’s ability to grasp and position items accurately, which is crucial in tasks requiring fine motor control (Ackerman, 1988). The time required to complete the pick and place test is another important factor in assessing efficiency and hand-eye coordination in object handling. A shorter completion time indicates better object handling.

#### 3.2.1. Test setup

**Figure 6.**
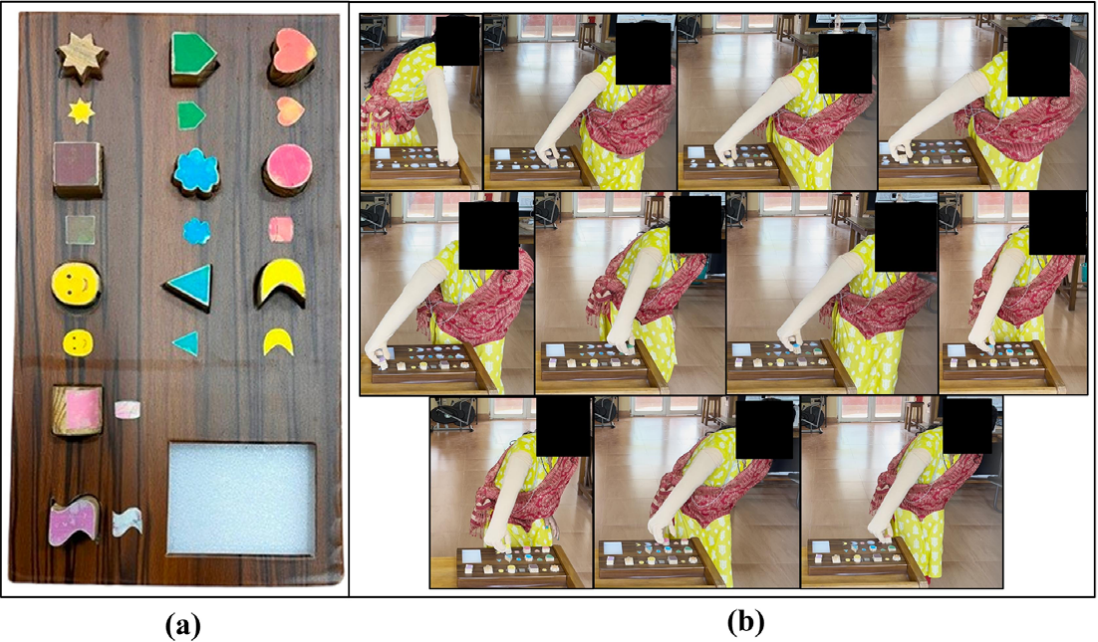
(a) Test setup for the pick and place test. (b) Eleven instances of trial T9 picking and placing each object correctly, during the pick and place test.

The pick and place test setup comprised 11 differently shaped objects as presented in Figure 3, and a test board with a corresponding hole is shown in Figure 6(a). The dimension of the test setup is presented in Table 3.

**Table 3.** Pick and place test setup dimensions (in cm)

	Length	Width	Height
Setup box	37.7	21.7	4
Holes	^a^	^a^	2
Blocks	^b^	^b^	3.4

a Holes lengths and width depend on the block lengths and width.

b Blocks lengths and widths are represented in Figure 3.

#### 3.2.2. Experiments

The subject preparation for the experiment is detailed in Section 3.1.2. For the pick and place test, the subject was instructed to stand by the test setup while wearing ENRICH. The 11 objects were placed on the side of the test board. The task given to the subject is to grasp each object one by one and place it into its corresponding hole on the test board. In a single trial for each object, the user has one attempt to place it in the corresponding hole. If the subject failed on the first attempt, it was considered a failed attempt.

The number of correctly placed items on the test board and the time taken for completing one trial were recorded. The experiment was conducted for 10 trials in two phases: five trials in Phase I and five trials in Phase II. The test is conducted on two experimental days, with day 2 after 10 days of day 1. Figure 6(b) shows the subject performing the pick and place test by picking up and placing each object in corresponding shaped places correctly.

#### 3.2.3. Experimental results

The results of the pick and place test were recorded in two categories: (a) number of items correctly placed during each trial and (b) time taken to complete a trial. The scores of the test are tabulated in Table 4.

**Table 4.** Pick and place test score on day 1 and day 2

Phase	Trial	Day 1		Day 2	
		Items correctly placed	Time taken to complete the task	Items correctly placed	Time taken to complete the task
Phase I	T1	5	3 min 51 s	9	3 min 02 s
	T2	7	3 min 47 s	9	2 m 41 s
	T3	8	3 min 01 s	8	2 min 30 s
	T4	7	3 min 39 s	10	2 min 27 s
	T5	7	2 min 58 s	9	2 min 33 s
30 min rest					
Phase II	T6	9	3 min 38 s	11	2 min 02 sec
	T7	5	3 min 13 s	10	2 min 11 s
	T8	9	3 min 21 s	11	2 min 05 s
	T9	11	3 min 02 s	11	2 min 03 s
	T10	9	3 min 14 s	10	1 min 50 s

Table 4 shows that on day 1, the median value of the number of items placed in Phase I was seven, which was improved to nine in Phase II. This improvement can be seen in day 2 as well, where the median values of items correctly placed in Phase I and Phase II are 9 and 11, respectively. Results of day 2 are also improved compared to that of day 1. Similarly, the time taken to complete the trials in Phase II is reduced compared to that of Phase I for both experimental days. Further, time taken on day 2 reduced significantly compared to day 1. This indicates clear improvement in the object handling capability of the subject using ENRICH. The results show ENRICH is capable of handling objects, which represents its usability in daily living activities. To present the results in a quantitative way, the pick and place test scores were expressed in seconds per item (sec/item), that is time taken to complete one trial per number of correctly placed items. It was estimated using Equation (3.2).



(3.2)

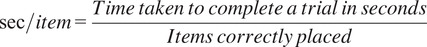



**Figure 7.**
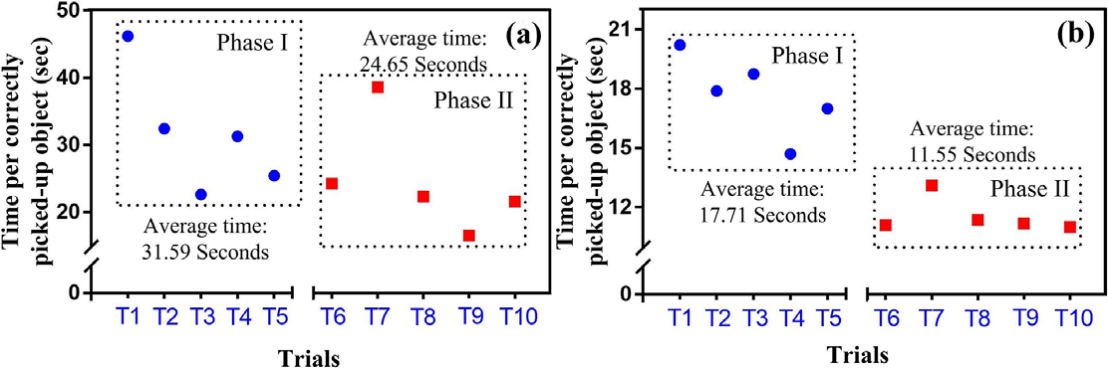
Time taken for each correctly placed object with respect to trials on (a) day 1 and (b) day 2.

The sec/item is directly proportional to the time taken by the user to complete a trial and inversely proportional to the number of items correctly placed. Therefore, a lesser value of sec/item indicates a better result. The sec/item in the pick and place test were presented in Figures 7(a) and (b) for day 1 and day 2, respectively. It shows that the performance of ENRICH improved with the increase in trials. The average sec/item on day 1 in Phase I was 31.59 sec/item which was improved to 24.65 sec/item, in Phase II. The improvement is significant on day 2 compared to day 1 with an average sec/item value of 17.71 sec/item and 11.55 sec/item in Phase I and Phase II, respectively. This improvement shown by ENRICH over time is because of the visual biofeedback discussed in Section 3.1.4. Over time, with consistent practice, this visual feedback system can refine performance, ensuring better object handling by ENRICH. The results of pick and place test ensure that ENRICH is well capable of performing daily living activities. With practice, the usability of ENRICH improved, and subjects could handle objects for their daily activities seamlessly. This fact is further validated in the pilot testing of ENRICH, where subjects used ENRICH in their daily living activities for a year.

## 4. Functional characteristics

### 4.1. Physical properties

#### 4.1.1. Size

The human hand lengths from wrist to distal phalanx of the middle finger ( 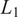 ), elbow to wrist 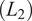 , and shoulder to elbow ( 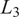 ) constitute 10%, 14.6%, and 18.6% respectively of human height (Panero et al., 1979). The typical height of an adult male is 178 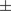 7 cm and for an adult female is 165 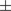 7 cm (Visscher, 2008). Therefore, typical hand length for an adult male is 77 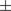 7 cm and for an adult female is 71 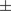 7 cm. Considering these as the basis of dimensions for ENRICH, lengths 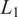 and 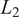 of ENRICH were set as given in Table 5 to mimic the human counterpart. The length of the respective sockets may be adjusted to match the length of the amputated hand from elbow to shoulder. Further, ENRICH maintains the ratio of distal to intermediate to proximal phalanges for index, middle and ring fingers as 1:1.3:2.3, little finger as 1:2:1, and the ratio of distal to proximal phalanges for the thumb as 1:1.4 (Hutchison and Hutchison, 2010; Ozsoy et al., 2019).

**Table 5.** Lengths of different parts of ENRICH vis-à-vis human hand

	Wrist to distal phalanx of the middle finger, 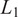 (cm)	Elbow to wrist 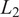 (cm)	Shoulder to elbow 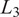 (cm)
Male	17.8	25.99	33.11
ENRICH (male)	17.8	15 + 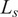 ^a^	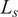 ^a^
Female	16.5	24.09	30.69
ENRICH (female)	16.5	15 + 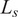 ^a^	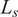 ^a^

a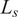 is the length of socket.

#### 4.1.2. Weight

Weights of the human fingers and palm ( 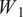 ), elbow to wrist ( 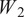 ), and shoulder to elbow ( 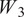 ) are 0.9%, 1.7%, and 3.2% respectively of the human body weight (Krishnan et al., 2016). The average weight of an adult is 70 kg (Dourson and Stara, 1983). The average weights of distinct sections of a human hand were estimated to be 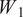 = 630 g, 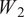 = 1,190 g, and 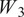 = 2,240 g. Users have reported that prosthetic hands with weight same as human hand are excessively heavy (Belter and Dollar, 2011). Therefore, weight of a prosthetic hand should be less than the human counterpart. Table 6 shows the weight of different parts of ENRICH vis-à-vis human hand.

**Table 6.** Weights of different parts of ENRICH vis-à-vis human hand

Parts	ENRICH (g)	Human hand (g)
Fingers and palm ( 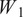 )	120	630
Elbow to wrist ( 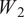 )	395	1,190
Shoulder to elbow ( 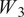 )	^a^	2,240
Socket for transhumeral amputees	130	^b^
Socket for transradial amputees	200	^b^

a Shoulder to elbow part of ENRICH is adjusted by the socket length.

b Not relevant.

#### 4.1.3. Interfacing socket

The traditional method of prosthetic hand design involves plaster or fiberglass cast in the residual limb to create a mold (Kempfer et al., 2022). The mold of the limb is created to design a custom-fitted socket. Traditional prostheses are normally attached to the amputated limb using a tight compression cup (Li and Felländer-Tsai, 2021). It produces an unpleasant interaction between prosthetic and amputated hands (Li and Felländer-Tsai, 2021). ENRICH handled this issue with anatomic suspension using volume adjustable belts. As a response by a user during the clinical testing, this allowed the user to give appropriate pressure on the amputated hand by the socket. Further, the volume-adjustable belts make it simple to put on and take off the prosthetic hand. Users can perform the donning and doffing of ENRICH using their healthy hand.

Table 7 presents prosthetic hand sockets with their fabrication method and material type. From Table 7, a shift of fabrication methods from traditional to 3D-printed methods was observed. Further, composite materials were chosen for the prosthetic hand socket design because of their ability to provide a balance of strength and durability (Current et al., 1999; Neo et al., 2000). However, with the adaptability of additive manufacturing methods, materials like carbon fiber (Türk et al., 2018; Nickel et al., 2020), PEGT (Owen and DesJardins, 2020), Polypropylene (Stenvall et al., 2020), PLA (Campbell et al., 2012; Owen and DesJardins, 2020) are commonly used. The interfacing socket for ENRICH was designed using additive manufacturing with PLA material. The inner part of the socket was filled with silicone for additional strength and softness while connecting to the residual limb.

**Table 7.** Comparison of different prosthetic hand sockets

Author	Year	Fabrication	Material type
Current et al. (1999)	1999	Traditional	Composite
Neo et al. (2000)	2001	Traditional	Composite
Graebner and Current (2007)	2007	Traditional	Composite
Campbell et al. (2012)	2012	3D printed and traditional	Composite and PLA
Gerschutz et al. (2012)	2012	Traditional	Composite
Türk et al. (2018)	2018	3D printed	Carbon fiber
Pousett et al. (2019)	2019	3D printed and traditional	Composite and PLA
Owen et al. (2020)	2020	3D printed and traditional	PETG and PLA
Nickel et al. (2020)	2020	3D printed	Carbon fiber
Stenvall et al. (2020)	2020	3D printed	Polypropylene
ENRICH	2024	3D Printed	PLA

### 4.2. Grasping properties

#### 4.2.1. Control strategy

ENRICH performs grasping operations by using EMG from the trapezius muscle. Single-channel Ag/AgCl surface electrodes were utilized to collect EMG. To avoid cumbersome while wearing multichannel (Gohain et al., 2022), single-channel electrodes EMG were considered in ENRICH. The differential inverting and non-inverting terminal electrodes were positioned on the longitudinal midline of the trapezius muscle, with the reference electrode located in the collarbone. An instrumentation amplifier amplifies EMG with a gain of 100 and a common mode rejection ratio of 110 dB. The collected EMG was pre-processed, and the extracted feature, that is root mean square (RMS), was fed into a finite state algorithm (FSA) for understanding users’ grasping intention. Initially, FSA output keeps ENRICH in a grasp open state. On recognition of grasping intention, FSA output changes ENRICH to the grasp close state. The details of the EMG control were reported in (Kakoty et al., 2022).

**Figure 8.**
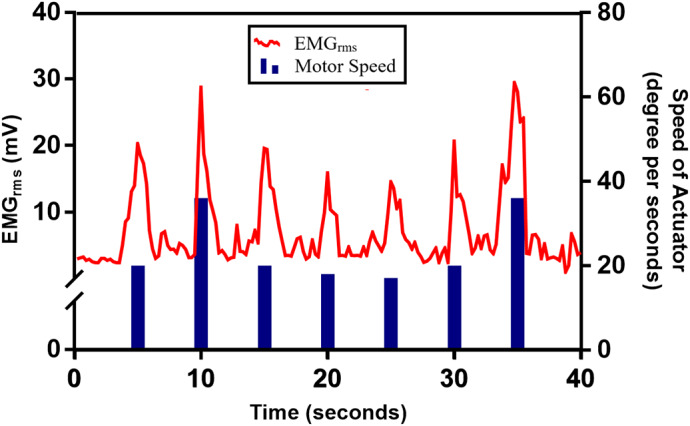
Proportional variation of actuator’s speed with respect to the EMG amplitude.

To provide intuitive grasping ability, proportional control was incorporated in ENRICH. Figure 8 shows the proportional variation in speed of the actuator with respect to the EMG amplitude. Proportional control in EMG-based prosthetic hands utilizes the amplitude of EMG generated by muscle to control the speed of the prosthetic hand. The amplitude of the EMG signal is directly proportional to the force of the muscle contraction (Li et al., 2014). The proposed control algorithm understands the processed RMS of EMG amplitude ( 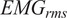 ) and translates them into proportional movements of the prosthetic hand by changing the speed of actuator.

#### 4.2.2. Grasping time

**Figure 9.**
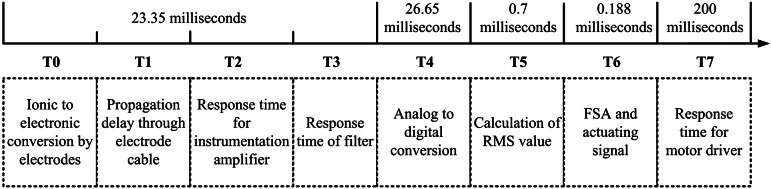
Operation time of ENRICH.

The estimated time necessary for the grasping task by ENRICH was divided into eight intervals (T0, T1, T2, T3, T4, T5, T6 and T7), as illustrated in Figure 9. Detailed information on the estimation of grasping time was reported in (Kakoty et al., 2022). The average grasping time was calculated to be 250.8 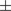 1.1 ms, fulfilling the neuromuscular constraints of the human hand (Kakoty et al., 2022). This speed mimics natural hand movements, ensuring that the prosthetic is responsive enough for real-time interaction with objects and not too fast, which gives a more robotic feeling rather than a counterpart of the human hand.

#### 4.2.3. Grasp force

**Figure 10.**
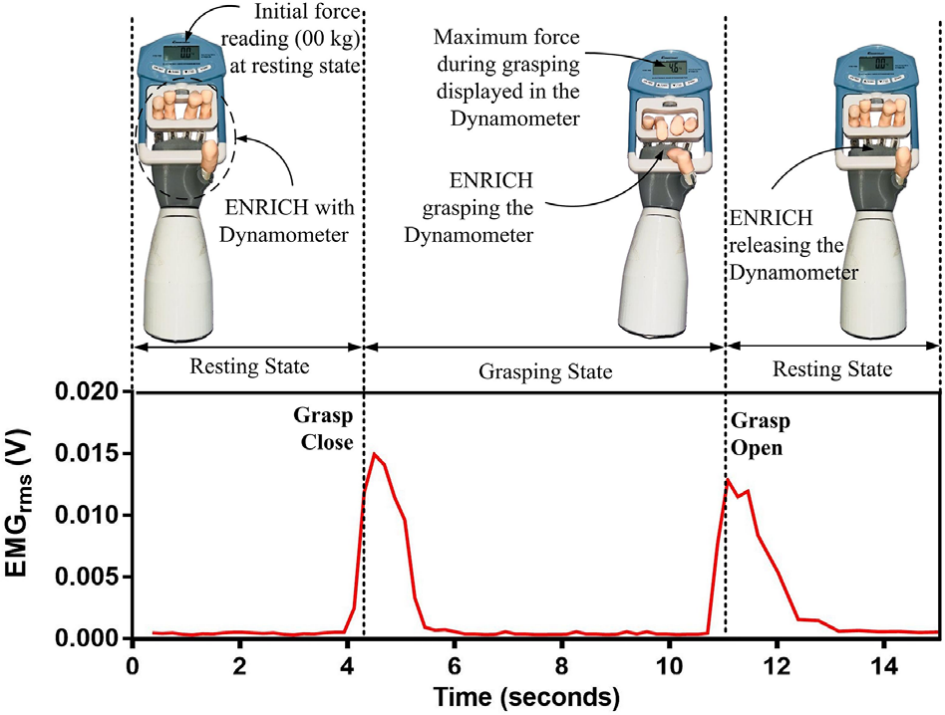
The experimental method for grasp force measurement of ENRICH using a hand Dynamometer.

**Figure 11.**
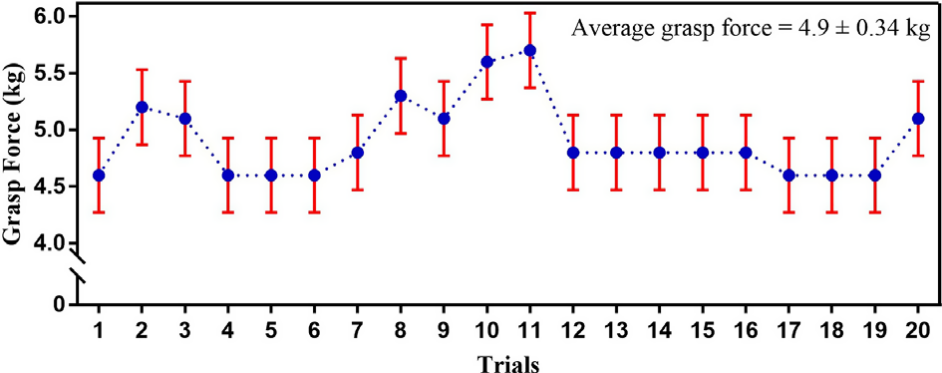
Grasp force of ENRICH estimated using a hand dynamometer.

Grasp force is an essential attribute for prosthetic hand functionality. Using a hand dynamometer for grasp force measurement by prosthetic hands is a well-established method (Polisiero et al., 2013; Mühldorfer-Fodor et al., 2014; Cuellar et al., 2019; Cuellar et al., 2021; Ramos et al., 2023). In the reported work, the power grasp force in ENRICH was determined by applying the force generated while grasping a hand dynamometer (EH101). The experimental testbed for grasp force measurement of ENRICH on recognition of EMG for grasping intention is shown in Figure 10. To measure grasp force, initially, ENRICH was kept in a resting state for 4 s. On recognition of grasp close intention based on EMG, ENRICH grasped the hand dynamometer handle and held it for 7 s. The placement of the dynamometer was successfully determined based on the movement of the prosthetic hand to ensure that optimal force was applied to the dynamometer. Following this, on recognition of grasp open intention, ENRICH releases the dynamometer’s handle. The dynamometer displayed the maximum force data applied during the grasping state. The experiment was conducted for 20 trials, and the recorded data are presented in Figure 11. The average grasp force found was 4.9 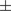 0.34 kg ( 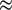 49 N). A comparison of the grasp force of different prosthetic hands vis-à-vis ENRICH is presented in Section 5.2, which indicates that ENRICH demonstrates adequate grasp forces for securely holding objects. This finding is further substantiated by the experimental results detailed in Section 2.1, which showed that no objects were dropped during the initial grasping tests conducted with ENRICH.

#### 4.2.4. Weight lifting capacity

ENRICH’s capacity for weight lifting was determined utilizing an experimental setup that included a water bottle, a 3D-printed handle, a weighing balance, and a working prototype of ENRICH. A video of the experiment is available at: *https://youtu.be/b9v_bLVi9og?si=.* When ENRICH recognizes the grasping intention of the user, it holds and lifts the handle where the water bottle was attached. This was done for ten trials. For each subsequent trial, the volume of water in the bottle was raised by 250.8 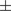 1.1 ms. ENRICH was able to lift the water bottle up to the ninth trial and slips on the 10th. The weight of the bottle and the handle was measured in a weighing balance after the ninth trial, and it was observed that ENRICH’s weight lifting capacity was 2,150 g.

### 4.3. Kinematic properties

#### 4.3.1. Degrees of freedom

DoF directly indicates the functionality of a prosthetic hand. In ENRICH, each finger has three links, with the thumb having two links. Each link is interconnected with the successive and previous link using a revolute joint. The total DoF possessed by ENRICH is 14 (=1 thumb 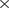 2 + 4 fingers 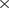 3).

#### 4.3.2. Range of motion

A Goniometer was used to estimate the RoM of the finger joint in ENRICH during flexion-extension. The joints of each finger were designed in a way that the rearward motion of the consecutive finger link was restricted, imitating a human finger. Table 8 represents the RoM of joints of ENRICH vis-à-vis the human counterpart. RoM indicates the functional range of the prosthetic hand required to perform daily living activities (Bain et al., 2015).

**Table 8.** RoM of different joints of ENRICH vis-à-vis human finger

	Human finger (Prudencio et al., 2014)	ENRICH finger	Human thumb (Barakat et al., 2013)	ENRICH thumb
MCP hyperextension	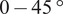	0	Not applicable	Not applicable
MCP flexion	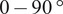	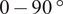	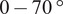	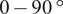
PIP flexion	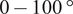	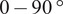	Not applicable	Not applicable
DIP flexion	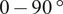	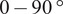	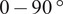	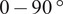

### 4.4. Discussions

The functional characteristics of the ENRICH are evaluated as physical, grasping, and kinematic properties. ENRICH is designed to mimic human anatomy with dimensions proportionate to average human hand measurements. The weight of ENRICH is lighter, enhancing its usability during daily living activities. The socket design utilizes 3D printing techniques. The anatomical suspension method uses a volume-adjustable belt, which provides an adjustable fit compared to the traditional sockets. Grasping operations in ENRICH are controlled via a single-channel EMG from the trapezius muscle, making movements intuitive with proportional control. The average grasping time of ENRICH is 250.8 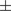 1.1 ms, fulfilling the neuromuscular constraints of the human hand. Because of the implementation of proportional control, the actuator speed of ENRICH can be varied with EMG intensity. Thus, the grasping time can be adjusted in ENRICH to users’ needs. The grasp force of ENRICH was estimated to be 49 N (approx) and the weight lifting capacity was 2,150 g. The DoF of ENRICH is 14, and the RoM of ENRICH is similar to the RoM of human fingers. This makes ENRICH’s functional range similar to its human counterpart. The functional characteristics of ENRICH highlight its design and usability. ENRICH takes a significant step in replicating the human hand by integrating anatomical dimensions comparable to an average human hand, a lightweight design, an adjustable socket, intuitive control mechanisms, a human-like functional range, and sufficient grasp force to perform daily living activities. These functional characteristics show ENRICH as a promising prosthetic hand, offering tangible advantages to amputees.

## 5. Comparison and analysis

The performance of ENRICH was compared with most of the widely referred research prototypes and market-dominant commercial prosthetic hands. Table 9 presents the physical and kinematic features; Tables 10 and 11 present grasping features of ENRICH in comparison to 10 commercial variants and 26 laboratory prototypes. Physical properties namely size and weight, as well as the kinematic properties, namely numbers of joints, numbers of actuators, and DoF, were considered. For grasping properties, number of EMG channels, grasping time, and grasp force were considered.

**Table 9.** Physical and kinematic properties of different prosthetic hands vis-à-vis ENRICH

Prosthetic hand	Physical properties		Kinematic properties		
	Length (mm)	Weight (g)	Numbers of joints	Numbers of actuators	DoF
Commercial prosthetic hands					
TASKA (Prosthetics, 2022)	197–210	556–644	Not available	6	8
BeBionic (Ottobock, 2020; Kakoty et al., 2022; Belter and Dollar, 2011)	198	495–539	11	5	11
i-Limb (Össur, 2021; Kakoty et al., 2022; Belter and Dollar, 2011)	180–182	450–650	11	5	11
Michelangelo Hand (Ottobock, 2024)	180	420	11	2	6
Zeus Hand (Biomedical, 2024)	172	560	11	5	10
Gifu Hand III (Mouri et al., 2002; Hand, 2024)	251.3	1400	20	10	16
Psyonic Ability Hand (PSYONIC, 2024)	Not available	490	11	5	11
Laboratory prototypes					
RTR hand II (Belter and Dollar, 2011)	Not available	350	9	2	9
Remadi Hand (Belter and Dollar, 2011)	Not available	400	14	6	6
MANUS Hand (Pons et al., 2004; Belter and Dollar, 2011)	Not available	1200	9	2	3
Galileo Hand (Fajardo et al., 2020)	184	350	15	6	15
UB Hand 3 (Belter and Dollar, 2011)	Not available	Not available	18	16	16
DLR/HIT II (Belter and Dollar, 2011)	Not available	1500	20	15	15
DLR/HIT I (Belter and Dollar, 2011)	Not available	2200	17	13	13
Fluid Hand III (Belter and Dollar, 2011)	Not available	400	8	1 pump and 5 valves	8
Smart Hand (Belter and Dollar, 2011)	Not available	520	16	4	16
Octa Hand (Abarca et al., 2019)	206	950	15	6	6
Keio Hand (Belter and Dollar, 2011)	320	730	15	1	15
TBM Hand (Belter and Dollar, 2011)	Not available	280	15	1	6
Vanderbilt Hand (Belter and Dollar, 2011)	190	580	16	5	16
PRISMA Hand II (Weiner et al., 2022)	210	336 (excluding controller)	14	3	19
KIT Prosthetic Hand (Weiner et al., 2022)	232	768	Not available	2	10
Modular Prosthetic Limb (Johannes et al., 2020)	Not available	4700	26	17	26
SSSA MyHand (Controzzi et al., 2016)	200	478	10	3	4
RIC Arm (Lenzi et al., 2016)	396	1518	10	1	5
ENRICH	Specified in Table 5	Specified in Table 6	14	2	14

**Table 10.** Number of EMG channels and grasping time of different prosthetic hand vis-à-vis ENRICH

Prosthetic hand	Number of EMG channels	Grasping time
Commercial prosthetic hands		
Michelangelo (Belter and Dollar, 2011; Gohain et al., 2022)	Multiple channels	Not available
Zeus hand (Biomedical, 2024)	Two channels	1.2 s
Bebionic (Wong et al., 2017; Ottobock, 2020; Gohain et al., 2022;)	2	0.8–1.9 s
i-limb (Belter and Dollar, 2011; Simon et al., 2019; Gohain et al., 2022;)	8	1.5 s
Psyonic ability hand (PSYONIC, 2024)	Multiple channels	200 ms
Mia hand (Prensilia – Grasping innovation, 2024)	Multiple channels	300 ms
Kal arm (Hive, 2023)	2	1 s
Indy hand (Motorica, 2024)	2	1.5 s
Laboratory prototypes		
SmartHand (Belter and Dollar, 2011; Gohain et al., 2022)	Not available	1.4 s
Galileo hand (Fajardo et al., 2020; Gohain et al., 2022)	2	Not available
Kyranou et al. (Belter and Dollar, 2011; Gohain et al., 2022)	12	Not available
Zhou et al. (Zhou et al., 2019; Gohain et al., 2022)	Multiple channels	Not available
MANUS-hand (Belter and Dollar, 2011; Gohain et al., 2022)	Not available	1.2 s
Remedi hand (Belter and Dollar, 2011; Gohain et al., 2022)	Not available	2.5 s
KIT prosthetic hand (Weiner et al., 2022)	Not available	0.73–1.32 s
SSSA MyHand (Controzzi et al., 2016)	Not available	270–370 ms
RIC Arm (Lenzi et al., 2016)	Not available	400 ms
Modular prosthetic limb (Johannes et al., 2020)	Multiple channels	300 ms
Zhang et al. (Zhang et al., 2016)	6	1 s
ENRICH	1	250.8 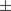 1.1 ms

### 5.1. Comparison based on eeight

Commercial variants of prosthetic hands weigh from 420 to 1,400 g, whereas laboratory prototypes range from 280 to 4,700 g. However, the majority of studies either do not specify or do not disclose the forearm’s weight. It was observed that prosthetic hands use one to sixteen actuators. The hand weights rise as the number of actuators increases. Compared to most prosthetic hands, ENRICH is lightweight, weighing 515 g with two actuators.

### 5.2. Comparison based on grasping properties

Prosthetic hands with multichannel EMG and grasping time are tabulated in Table 10. Most of the prosthetic hands use multichannel EMG-based control methodology. However, the user finds discomfort while wearing multiple EMG channels (Gohain et al., 2022). ENRICH stands out in this aspect by using a single-channel EMG for controlling grasping operation. Further, most prosthetic hands have grasping times between 0.8 and 2.5 s (Belter and Dollar, 2011; Wong et al., 2017; Ottobock, 2020; Weiner et al., 2022). On the contrary, ENRICH takes 250.8 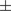 1.1 ms from the initiation of the user’s intent to grasping an item (Kakoty et al., 2022). Only a few hands (Psyonic Ability Hand, Mia Hand, SSSA MyHand and Modular Prosthetic Limb) along with ENRICH have grasping time comparable to the human hand.

The comparison of grasp force between various prosthetic hands vis-à-vis ENRICH is presented in Table 11. Prosthetic hands such as the MANUS Hand, Bebionic V2, SmartHand, and i-limb demonstrate high grasp forces during power grasping, ranging from 60 N to 136 N. In contrast, devices like CyberHand, RTR Hand II, and hand reported by Zhang et al. (Zhang et al., 2016) and PrHand1 show lower forces, producing 5 N, 15 N, 20 N, and 13.38 N, respectively. ENRICH’s grasp force is lower than some of the prosthetic hands but exceeds that of a few others. This comparison indicates that CyberHand, RTR Hand II, and hand reported by Zhang et al. (Zhang et al., 2016) and PrHand offer progressively lower grasp force, making them more suitable for precision or delicate tasks. These are better suited for specialized, low-force applications. Prosthetic hands like i-limb and SmartHand offer more than 100 N grasp force, making them good for tasks that require more grasp force. However, Bebionic V2, MANUS Hand, PrHand2, and ENRICH present enough grasp force that can hold most objects required to perform daily living activities (Rice et al., 1998).

**Table 11.** Comparison of grasp forces of different prosthetic hands vis-à-vis ENRICH

Prosthetic hand/author	Grasp force
CyberHand (Carrozza et al., 2006 ; Zhang et al., 2016)	5 N
RTR Hand II (Takayama et al., 2010; Zhang et al., 2016;)	15 N
Zhang et al. (Zhang et al., 2016)	20 N
PrHand1 (Ramos et al., 2023)	23.38 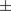 1.5 N
PrHand2 (Ramos et al., 2023)	36.13 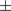 2.3 N
MANUS Hand (Gaiser et al., 2009; Zhang et al., 2016;)	60 N
Bebionic V2 (Zhang et al., 2016)	75 N
SmartHand (Cipriani et al., 2011 ; Zhang et al., 2016;)	100 N
i-limb (Zhang et al., 2016)	136 N
ENRICH	4.9 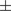 0.34 kg ( ≈ 49 N)

### 5.3. Comparison based on kinematic properties

The DoF and number of actuators for the prosthetic hands under study are presented in Table 9. It was observed that the hand does not need additional actuators when utilizing underactuation to produce higher DoF. Higher DoF is demonstrated by the Keio Hand, Velderbit Hand, Galileo Hand, and ENRICH with fewer actuators.

### 5.4. Comparison based on clinical testing

**Figure 12.**
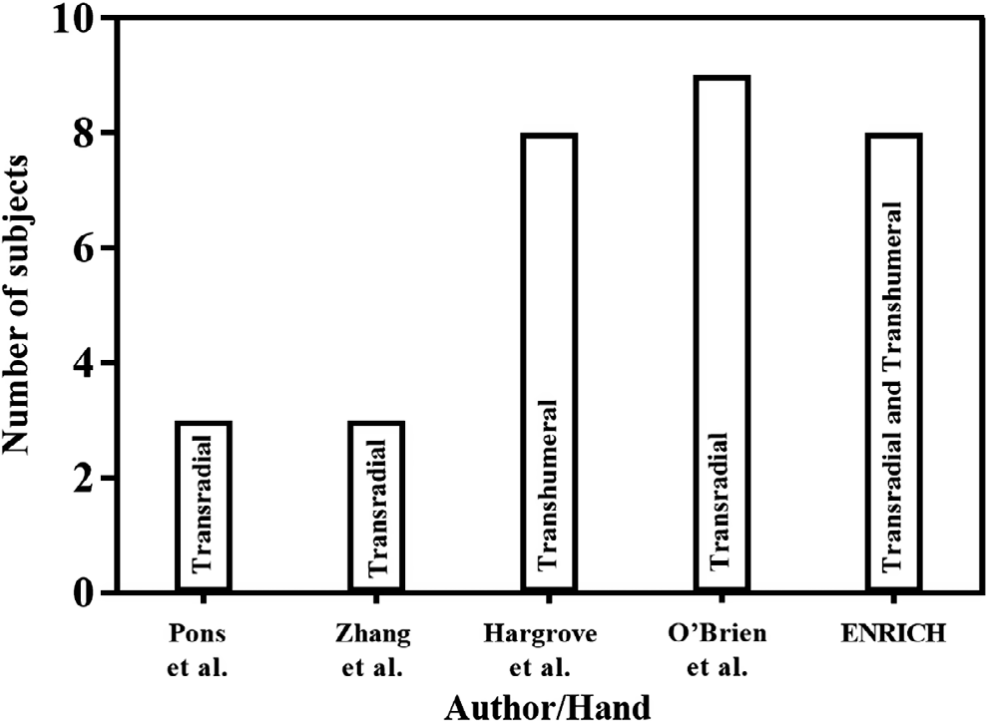
Test subjects for clinical testing considered by Pons et al. (2004), Zhang et al. (2011), Hargrove et al. (2017) and O’Brien et al. (2022).

According to the work reported in (Østlie et al., 2012), various types of hand amputation, that is transradial, transhumeral, and congenital amputation, were included in clinical testing. Further, a preliminary clinical examination of upper limb prosthesis can be completed with six or seven subjects (Wijk et al., 2020). In this study, eight subjects (three female and five male) were considered for the clinical testing of ENRICH. Amputations were caused by burning (2), electric shock (1), road accidents (4), and congenital (1) reasons. This represents that all kinds of hand amputations were considered in the clinical testing. Following the clinical testing, a pilot test that is the use of ENRICH for a longer duration (one year) during daily living activities, was accomplished with three amputees. The comparison of the number of individuals and amputation types between ENRICH and other prosthetic hands (Pons et al., 2004; Zhang et al., 2011; Hargrove et al., 2017; O’Brien et al., 2022) is shown in Figure 12. Most of the studies were in lab settings and focused on a single type of amputation. However, ENRICH was evaluated with eight different types of amputation. Moreover, the assessment of functionality and usability through the BBT and, pick and place tests was conducted. Additionally, pilot testing in a real-world environment validates user acceptability, which is limited in most previously reported work.

### 5.5. Discussions

A comparison of ENRICH vis-à-vis 10 commercial variants and 26 laboratory prototypes was made based on weight, grasping properties, kinematic properties, and clinical testing. Even if some prosthetic hands do not have user trials to support their performance, there is sufficient reported data on these prosthetic hands to demonstrate their performance. Therefore, a comprehensive comparison was considered for various attributes that are vital for a prosthetic hand. It was observed that most of the available prosthetic hands use multiple EMG channels, whereas ENRICH was controlled by a single-channel EMG. The use of a single-channel strategy enables ENRICH to perform grasping within 250.8 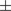 1.1 ms. Very few prosthetic hands, namely SSSA-Myhand, Prensilia MIA Hand, and the Modular Prosthetic Limb, have grasping time satisfying neuromuscular constraints. However, these hands are not reported for their clinical experiments and are mostly evaluated in a constrained laboratory environment (Controzzi et al., 2016; Johannes et al., 2020). Other prosthetic hands’ grasping time is in the order of seconds. The use of a single channel also contributes to reduce computational costs, which leads to weight reduction. The comparison of weight reveals that commercial prosthetic hands typically range between 420 and 1,400 g (Mouri et al., 2002; Ottobock, 2024; Hand, 2024), while laboratory prototypes range from 280 to 4,700 g (Belter and Dollar, 2011; Weiner et al., 2022). ENRICH weighing 515 g presents a lightweight solution to amputees.

The comparison of kinematic properties unveils that ENRICH, along with prosthetic hands namely Velderbit Hand, Keio Hand, and Galileo Hand, achieve higher DoF with fewer actuators. The under-actuated prosthetic hand with 14 passive DoFs shows excellent adaptation to grasp objects of various shapes as demonstrated in the pick and place test. The analysis of grasp force demonstrates that ENRICH delivers a grasp force comparable to that of many prosthetic hands. Its ability to carry a weight of 2 kg indicates that it can effectively grasp and handle most objects necessary for performing daily living activities.

One of the major contributions of the reported work was the design improvement based on clinical testing for more usability and user acceptability. The clinical testing involved eight subjects with different types of hand amputations. The BBT and pick and place test confirm that ENRICH’s performance and usability improve with practice over time. Visual feedback for slippage prevention assisted to achieve ENRICH’s performance improvement. Also, pilot testing was carried out with three amputees for one year, where the subjects could perform their daily living activities using ENRICH. The pilot testing also demonstrated effective integration between the user’s residual limb and the prosthetic hand, facilitated by the socket, which enabled them to perceive the motion of the hand as it moved over the object. The users’ feedback during pilot testing was also used to improve ENRICH and was considered an important part of the development process. These considerations differentiate ENRICH from studies predominantly conducted, as very few reports have addressed these aspects.

It is observed that numerous studies emphasized the capabilities of their reported prosthetic hands without adequately assessing usability. As a result, many prosthetic hands excel in specific attributes but lack comprehensive usability evaluations. This study addresses this gap by conducting tests such as BBT, pick and place tasks, and subsequent pilot testing to ensure both the functionality and usability of ENRICH.

In summary, the comprehensive functional assessment demonstrates that while some prosthetic hands perform exceptionally well in one or two key attributes, ENRICH’s performance is comparable or better across all critical functional attributes. Thereby, ENRICH offers a highly promising and practical solution for amputees.

## 6. Conclusions

To increase the prosthetic hand’s acceptance by users, evaluation of functional characteristics is crucial. The study presented in this manuscript evaluated the functional characteristics of a real-time EMG-controlled prosthetic hand called ENRICH. Physical, grasping and kinematic properties of ENRICH were evaluated in comparison to the human hand. The study reported clinical testing as one of the significant methods to evaluate the functional characteristics of prosthetic hands. Clinical testing of ENRICH was conducted with eight subjects following pilot testing with three subjects. ENRICH’s functionality and usability were assessed using the BBT and pick and place tests, which are indicative of activities of daily living. These assessments showed that with an increase in the number of trials, the performance of ENRICH gets better. The benchmark data presented in this study provide valuable insights for conducting clinical testing of EMG-controlled prosthetic hands in real-world scenarios. The functional characteristics evaluation indicates that ENRICH is lightweight, comparable in size to an average human hand, features an adjustable socket design, and offers intuitive control with a human-like range of motion. Functional attributes of ENRICH were compared with 10 commercial variants of prosthetic hands (TASKA Hand, Bebionic, Michelangelo Hand, Zeus Hand, Gifu Hand III, Psyonic Ability Hand, Mia Hand, Kal Arm, Indy Hand and i-Limb) and 26 laboratory prototypes (Remadi Hand, TBM Hand, MANUS Hand, UB Hand 3, DLR/HIT II, DLR/HIT I, FluidHand III, Galileo Hand, SmartHand, Octa Hand, Vanderbilt Hand, PRISMA Hand II, KIT Prosthetic Hand, RTR hand II, Modular Prosthetic Limb, RIC Arm, Keio Hand, SSSA MyHand, CyberHand, PrHand1, PrHand2 and Prosthetic hand reported by Kyranou et al. (Belter and Dollar, 2011), Zhou et al. (2019), Zhang et al. (2011), O’Brien et al. (2022) and Hargrove et al. (2017)). According to this assessment and comparison, ENRICH is one of the most promising prosthetic hands to restore functionality to hand amputees. Evaluation of ENRICH in terms of prosthetic hand assessment protocols like ACMC is part of ongoing work.

## Data Availability

All necessary data are included in the manuscript.
